# Redetermination of μ-oxido-bis­[bis­(*N*,*N*-diethyl­hydroxyl­aminato)­oxido­vanadium(V)]

**DOI:** 10.1107/S1600536811020551

**Published:** 2011-06-04

**Authors:** Heng-Qiang Zhang, Zhong-Min Jin, Xin Fan, Qi-Ying Zhang

**Affiliations:** a Department of Chemistry, East China Normal University, Shanghai 200062, People’s Republic of China

## Abstract

In comparison with the previous determination [Saussine, Mimoun, Mitschler & Fisher (1980 ▶). *Nouv. J. Chim.* **4**, 235–237] of the title compound, [V_2_(C_4_H_10_NO)_4_O_3_], the current study reports an improved precision of the derived geometric parameters, along with the deposition of all coordinates and displacement parameters. The two V^V^ atoms are each surrounded by two deprotonated *N*,*O*-bidentate diethyl­hydroxy­laminate groups, and a terminal and a bridging oxide ligand, in a distorted octa­hedral coordination geometry. The crystal packing is accomplished by van der Waals inter­actions.

## Related literature

For the previous determination, see: Saussine *et al.* (1980 ▶). For the pharmacological activities of vanadium complexes, see: Posner *et al.* (1994 ▶); Zhou *et al.* (2000 ▶); Huyer *et al.* (1997 ▶); Nxumalo *et al.* (1998 ▶). For related hydroxyl­amide complexes, see: Zhang *et al.* (2009 ▶, 2010 ▶); Paul *et al.* (1997 ▶); Wieghardt *et al.* (1981 ▶). For van der Waals radii, see: Bondi (1964 ▶).
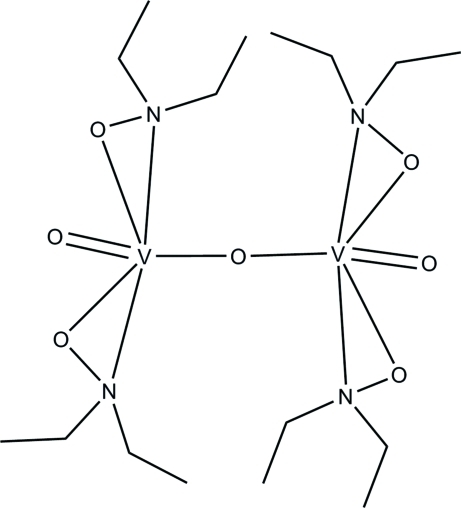


## Experimental

### 

#### Crystal data

[V_2_(C_4_H_10_NO)_4_O_3_]
*M* *_r_* = 502.40
Monoclinic, 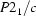

*a* = 14.6106 (3) Å
*b* = 10.2624 (2) Å
*c* = 19.4547 (3) Å
β = 120.744 (1)°
*V* = 2507.07 (8) Å^3^
*Z* = 4
Mo *K*α radiation
μ = 0.78 mm^−1^
*T* = 296 K
0.32 × 0.28 × 0.26 mm

#### Data collection

Bruker APEXII CCD diffractometer
28459 measured reflections
4419 independent reflections
3767 reflections with *I* > 2σ(*I*)
*R*_int_ = 0.028

#### Refinement

*R*[*F* ^2^ > 2σ(*F* ^2^)] = 0.030
*wR*(*F* ^2^) = 0.090
*S* = 1.05
4418 reflections
270 parameters
H-atom parameters constrained
Δρ_max_ = 0.28 e Å^−3^
Δρ_min_ = −0.15 e Å^−3^

### 

Data collection: *SMART* (Bruker, 2001 ▶); cell refinement: *SAINT-Plus* (Bruker, 2003 ▶); data reduction: *SAINT-Plus*; program(s) used to solve structure: *SHELXTL* (Sheldrick, 2008 ▶); program(s) used to refine structure: *SHELXTL*; molecular graphics: *XP* in *SHELXTL*; software used to prepare material for publication: *SHELXTL*.

## Supplementary Material

Crystal structure: contains datablock(s) I, global. DOI: 10.1107/S1600536811020551/wm2493sup1.cif

Structure factors: contains datablock(s) I. DOI: 10.1107/S1600536811020551/wm2493Isup2.hkl

Additional supplementary materials:  crystallographic information; 3D view; checkCIF report

## Figures and Tables

**Table 1:** Comparison of bond lengths (Å) and angles (°) between the previous determination (Saussine *et al.*, 1980 ▶) and the current study

Bond lengths	Reported	This work	Bond angles	Reported	This work
V1—N1	2.079 (4)	2.0906 (16)	O1—V1—N1	41.1 (1)	41.01 (6)
V1—N2	2.061 (4)	2.0797 (16)	O1—V1—O2	83.4 (1)	83.41 (6)
V1—O1	1.851 (3)	1.8726 (14)	O3—V2—O4	83.3 (1)	83.15 (6)
V1—O2	1.873 (3)	1.8790 (14)	O5—V1—O7	117.5 (1)	118.13 (7)
V1—O5	1.805 (3)	1.8139 (11)	N1—V1—N2	165.5 (1)	165.33 (7)
V1—O7	1.599 (3)	1.6012 (15)	V1—O5—V2	154.3 (1)	154.12 (8)
O1—N1	1.398 (5)	1.403 (2)	O5—V2—O6	117.6 (1)	117.86 (7)
O2—N2	1.400 (5)	1.413 (2)	O2—V1—N2	41.3 (1)	41.43 (6)
O3—N3	1.409 (5)	1.408 (2)	O3—V2—N3	41.2 (1)	41.37 (6)
O4—N4	1.402 (5)	1.408 (2)	O4—V2—N4	41.2 (1)	41.02 (6)

**Fig. 1.**
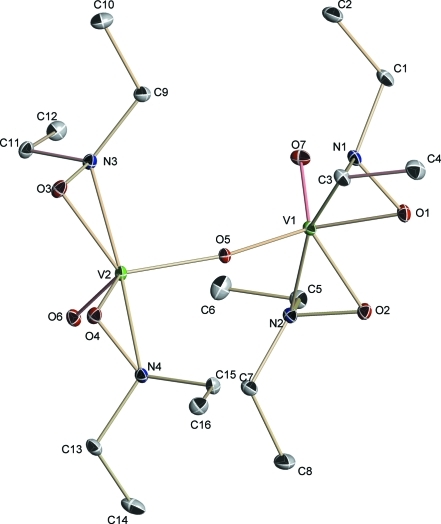
The molecular structure of the title compound with displacement parameters shown at the 30% probability level.

## supplementary crystallographic information

### Comment

The crystal structure of the title compound, [(VO(C_4_H_10_NO)_2_)_2_O], was first reported by Saussine *et al.* (1980). However, because atomic coordinates and displacement parameters have not been deposited (or are available) with the previous study, it is of interest to the public domain that this structure has been re-determined and to have access to the fully reported data.

Peroxidovanadium complexes are good insulin-mimetic compounds (Posner *et al.*, 1994; Zhou *et al.*, 2000). Studies suggest that the insulin-mimetic properties of peroxidovanadates are related to its oxidation at an active-site cysteine of the phosphatase (PTPs) that negatively regulate insulin receptor activation and signaling (Huyer *et al.*, 1997). Hydroxylamine is related to hydrogen peroxide and it forms some complexes with vanadium that are structurally similar to those formed with hydrogen peroxide. It is also reported that the vanadium-hydroxylamine complex, bis(*N,N*-dimethylhydroxamido)hydroxooxovanadate (DMHAV), is a potent inhibitor of the protein tyrosine phosphatase-1B (PTP1B), and that this inhibition does not involve an oxidative process. Molecular modelling studies suggest that the main stabilizing interaction of DMHAV in PTP1B are a cyclic H-bonded structure involving the conserved active site aspartate and hydrophobic stabilization interactions with the methyl groups of DMHAV (Nxumalo *et al.*, 1998). To gain further insight into the insulin mimetic actions of hydroxylamine complex, we have synthesized a group of vanadium-hydroxylamine complexes, including vanadium-aminoacids and vanadium-carboxylic acid hydroxylamido complexes (Zhang *et al.*, 2009; 2010)2. Here we report the synthesis and the redetermination of the structure of the title compound, [(VO(C_4_H_10_NO)_2_)_2_O]. The title compound was synthesized from ammonium metavanadate, DL-valine and sodium hydroxide. Compared to reported synthetic steps, the use of an aqueous reaction system and the vanadium source all simplifies the synthesis procedure; DL-valine may play a buffer role.

The molecular structure is shown in Fig. 1. In the crystal, no intermolecular separations significantly less than the sums of the appropriate van der Waals radii (Bondi, 1964) are found. The two vanadium atoms are six-coordinate within a considerably distorted octahedral coordination geometry defined by two deprotonated *N*,*O*-bidentate diethylhydroxylamine groups, an terminal and a bridging oxide ligand. In order to compare the difference of the previous determination and our work, some important bond length and bond angles are listed in Table 1.

A structurally similar dimethylhydroxamidovanadium(V) complex was previously prepared in a nonaqueous solvent system (Paul *et al.*, 1997; Wieghardt *et al.*, 1981).

### Experimental

To a solution of sodium hydroxide (0.2390 g,5.975 mmol) in H_2_O (10 ml), ammonium metavanadate (0.2142 g,1.831 mmol) and DL-valine were added under stirring. The resulting colorless solution was stirred for approximately two minutes in an ice bath. 2 ml of *N*,*N*-diethylhydroxylamine (25.9 mmol) were added dropwise. The mixture was stirred for approximately five minutes, and after filtration of the solution, yellow crystals were obtained by slow evaporation of a mixture of the filtrate and ethanol at 277 K over a period of a few days.

### Refinement

H atoms were placed in calculated positions, with C—H = 0.93 Å for phenyl, 0.96 Å for methyl and 0.97 Å for methylene H atoms, and refined as riding, with *U*_iso_(H) = 1.2*U*_eq_(C) for phenyl and methylene H, and 1.5*U*_eq_(C) for methyl H atoms.

### Crystal data

**Table:**

[V_2_(C_4_H_10_NO)_4_O_3_]	*F*(000) = 1064.0
*M**_r_* = 502.40	*D*_x_ = 1.331 Mg m^−^^3^
Monoclinic, *P*2_1_/*c*	Mo *K*α radiation, λ = 0.71073 Å
Hall symbol: -P 2y b c	Cell parameters from 9966 reflections
*a* = 14.6106 (3) Å	θ = 2.3–27.3°
*b* = 10.2624 (2) Å	µ = 0.78 mm^−^^1^
*c* = 19.4547 (3) Å	*T* = 296 K
β = 120.744 (1)°	Block, yellow
*V* = 2507.07 (8) Å^3^	0.32 × 0.28 × 0.26 mm
*Z* = 4	

### Data collection

**Table:**

Bruker APEXII CCD diffractometer	3767 reflections with *I* > 2σ(*I*)
Radiation source: fine-focus sealed tube	*R*_int_ = 0.028
graphite	θ_max_ = 25.0°, θ_min_ = 1.6°
φ and ω scans	*h* = −17→17
28459 measured reflections	*k* = −12→12
4419 independent reflections	*l* = −23→22

### Refinement

**Table:**

Refinement on *F*^2^	Primary atom site location: structure-invariant direct methods
Least-squares matrix: full	Secondary atom site location: difference Fourier map
*R*[*F*^2^ > 2σ(*F*^2^)] = 0.030	Hydrogen site location: inferred from neighbouring sites
*wR*(*F*^2^) = 0.090	H-atom parameters constrained
*S* = 1.05	*w* = 1/[σ^2^(*F*_o_^2^) + (0.0563*P*)^2^ + 0.3492*P*] where *P* = (*F*_o_^2^ + 2*F*_c_^2^)/3
4418 reflections	(Δ/σ)_max_ = 0.001
270 parameters	Δρ_max_ = 0.28 e Å^−^^3^
0 restraints	Δρ_min_ = −0.15 e Å^−^^3^

### Special details

**Table:**

Geometry. All e.s.d.'s (except the e.s.d. in the dihedral angle between two l.s. planes) are estimated using the full covariance matrix. The cell e.s.d.'s are taken into account individually in the estimation of e.s.d.'s in distances, angles and torsion angles; correlations between e.s.d.'s in cell parameters are only used when they are defined by crystal symmetry. An approximate (isotropic) treatment of cell e.s.d.'s is used for estimating e.s.d.'s involving l.s. planes.
Refinement. Refinement of *F*^2^ against ALL reflections. The weighted *R*-factor *wR* and goodness of fit *S* are based on *F*^2^, conventional *R*-factors *R* are based on *F*, with *F* set to zero for negative *F*^2^. The threshold expression of *F*^2^ > σ(*F*^2^) is used only for calculating *R*-factors(gt) *etc*. and is not relevant to the choice of reflections for refinement. *R*-factors based on *F*^2^ are statistically about twice as large as those based on *F*, and *R*-factors based on ALL data will be even larger.

### Fractional atomic coordinates and isotropic or equivalent isotropic displacement parameters (Å^2^)

**Table:**

	*x*	*y*	*z*	*U*_iso_*/*U*_eq_	
C1	0.26506 (19)	1.1932 (2)	0.82864 (15)	0.0639 (6)	
H1A	0.2345	1.1426	0.8541	0.077*	
H1B	0.2363	1.2808	0.8206	0.077*	
C2	0.3832 (2)	1.1991 (3)	0.88316 (17)	0.0826 (8)	
H2A	0.4109	1.1123	0.8983	0.124*	
H2B	0.3997	1.2482	0.9301	0.124*	
H2C	0.4148	1.2404	0.8560	0.124*	
C3	0.27143 (18)	1.2033 (2)	0.70312 (14)	0.0545 (5)	
H3A	0.2448	1.1591	0.6523	0.065*	
H3B	0.3486	1.1985	0.7318	0.065*	
C4	0.2383 (2)	1.3452 (2)	0.68728 (18)	0.0807 (8)	
H4A	0.1632	1.3521	0.6670	0.121*	
H4B	0.2541	1.3797	0.6486	0.121*	
H4C	0.2765	1.3938	0.7362	0.121*	
C5	0.02455 (18)	0.7146 (2)	0.70800 (15)	0.0607 (6)	
H5A	−0.0506	0.6964	0.6727	0.073*	
H5B	0.0320	0.7660	0.7524	0.073*	
C6	0.0836 (2)	0.5883 (3)	0.7393 (2)	0.0896 (9)	
H6A	0.0691	0.5326	0.6953	0.134*	
H6B	0.0606	0.5463	0.7720	0.134*	
H6C	0.1586	0.6053	0.7707	0.134*	
C7	0.05033 (17)	0.7317 (2)	0.59107 (14)	0.0570 (5)	
H7A	0.0935	0.6535	0.6053	0.068*	
H7B	0.0772	0.7919	0.5671	0.068*	
C8	−0.0630 (2)	0.6967 (3)	0.52945 (18)	0.0905 (9)	
H8A	−0.0894	0.6336	0.5515	0.136*	
H8B	−0.0651	0.6607	0.4831	0.136*	
H8C	−0.1066	0.7735	0.5145	0.136*	
C9	0.45814 (17)	0.8816 (2)	0.84272 (13)	0.0573 (5)	
H9A	0.4181	0.8633	0.8689	0.069*	
H9B	0.4295	0.9609	0.8119	0.069*	
C10	0.5730 (2)	0.9054 (3)	0.90619 (16)	0.0805 (8)	
H10A	0.6016	0.8288	0.9387	0.121*	
H10B	0.5771	0.9774	0.9391	0.121*	
H10C	0.6134	0.9250	0.8811	0.121*	
C11	0.47814 (17)	0.6443 (2)	0.82376 (13)	0.0568 (5)	
H11A	0.5543	0.6477	0.8611	0.068*	
H11B	0.4652	0.5819	0.7823	0.068*	
C12	0.4235 (2)	0.5990 (3)	0.86699 (16)	0.0735 (7)	
H12A	0.4452	0.6525	0.9133	0.110*	
H12B	0.4426	0.5100	0.8833	0.110*	
H12C	0.3478	0.6054	0.8321	0.110*	
C13	0.24236 (19)	0.7368 (2)	0.48238 (14)	0.0595 (6)	
H13A	0.2877	0.6620	0.5083	0.071*	
H13B	0.2588	0.7694	0.4431	0.071*	
C14	0.1285 (2)	0.6940 (3)	0.44040 (17)	0.0908 (10)	
H14A	0.1117	0.6613	0.4789	0.136*	
H14B	0.1176	0.6265	0.4028	0.136*	
H14C	0.0832	0.7667	0.4126	0.136*	
C15	0.21781 (18)	0.9668 (2)	0.51072 (13)	0.0513 (5)	
H15A	0.2298	1.0226	0.5548	0.062*	
H15B	0.1415	0.9550	0.4767	0.062*	
C16	0.2593 (2)	1.0349 (2)	0.46331 (15)	0.0652 (6)	
H16A	0.3355	1.0403	0.4949	0.098*	
H16B	0.2298	1.1211	0.4496	0.098*	
H16C	0.2389	0.9866	0.4153	0.098*	
N1	0.23255 (12)	1.13458 (16)	0.74962 (10)	0.0459 (4)	
N2	0.06387 (12)	0.79060 (15)	0.66428 (10)	0.0449 (4)	
N3	0.44108 (12)	0.77385 (16)	0.78718 (10)	0.0457 (4)	
N4	0.26690 (12)	0.83933 (16)	0.54295 (9)	0.0428 (4)	
O1	0.12126 (11)	1.12294 (14)	0.70484 (10)	0.0552 (4)	
O2	0.01895 (10)	0.91675 (14)	0.64920 (9)	0.0531 (4)	
O3	0.48410 (11)	0.80520 (15)	0.73892 (9)	0.0549 (4)	
O4	0.37801 (10)	0.85159 (14)	0.59304 (8)	0.0507 (3)	
O5	0.24996 (9)	0.90343 (12)	0.67831 (7)	0.0382 (3)	
O6	0.30604 (12)	0.64364 (13)	0.64972 (9)	0.0542 (4)	
O7	0.20071 (12)	0.90576 (16)	0.80668 (9)	0.0600 (4)	
V1	0.16510 (2)	0.94873 (3)	0.717301 (19)	0.03928 (11)	
V2	0.33748 (2)	0.79430 (3)	0.665006 (18)	0.03807 (11)	

### Atomic displacement parameters (Å^2^)

**Table:**

	*U*^11^	*U*^22^	*U*^33^	*U*^12^	*U*^13^	*U*^23^
C1	0.0742 (15)	0.0660 (15)	0.0695 (16)	−0.0132 (12)	0.0496 (13)	−0.0276 (12)
C2	0.0823 (18)	0.095 (2)	0.0673 (17)	−0.0157 (15)	0.0358 (15)	−0.0339 (15)
C3	0.0642 (13)	0.0473 (12)	0.0655 (14)	−0.0118 (10)	0.0430 (12)	−0.0099 (10)
C4	0.101 (2)	0.0492 (14)	0.089 (2)	−0.0061 (13)	0.0461 (17)	−0.0059 (13)
C5	0.0549 (12)	0.0666 (15)	0.0749 (16)	−0.0152 (11)	0.0434 (12)	−0.0038 (12)
C6	0.0785 (18)	0.088 (2)	0.111 (2)	0.0012 (15)	0.0552 (18)	0.0345 (18)
C7	0.0551 (12)	0.0623 (14)	0.0582 (14)	−0.0086 (10)	0.0322 (11)	−0.0148 (11)
C8	0.0680 (17)	0.107 (2)	0.0767 (19)	−0.0148 (15)	0.0226 (14)	−0.0346 (17)
C9	0.0568 (13)	0.0577 (13)	0.0517 (13)	−0.0029 (10)	0.0236 (10)	−0.0025 (10)
C10	0.0683 (16)	0.093 (2)	0.0618 (16)	−0.0159 (14)	0.0202 (13)	−0.0006 (14)
C11	0.0548 (12)	0.0564 (13)	0.0530 (13)	0.0133 (10)	0.0232 (10)	0.0145 (10)
C12	0.0768 (16)	0.0700 (16)	0.0752 (18)	−0.0056 (13)	0.0400 (14)	0.0153 (13)
C13	0.0819 (16)	0.0566 (13)	0.0534 (13)	0.0070 (11)	0.0443 (12)	−0.0048 (10)
C14	0.104 (2)	0.110 (2)	0.0738 (19)	−0.0393 (18)	0.0572 (18)	−0.0425 (17)
C15	0.0622 (13)	0.0507 (12)	0.0485 (12)	0.0118 (10)	0.0336 (10)	0.0089 (9)
C16	0.0760 (15)	0.0692 (16)	0.0545 (14)	0.0012 (12)	0.0363 (12)	0.0151 (11)
N1	0.0460 (9)	0.0460 (9)	0.0570 (10)	−0.0054 (7)	0.0344 (8)	−0.0132 (8)
N2	0.0414 (8)	0.0466 (9)	0.0529 (10)	−0.0041 (7)	0.0287 (8)	−0.0039 (7)
N3	0.0451 (9)	0.0504 (10)	0.0449 (9)	0.0065 (7)	0.0254 (8)	0.0084 (7)
N4	0.0476 (9)	0.0447 (9)	0.0434 (9)	0.0063 (7)	0.0286 (8)	0.0018 (7)
O1	0.0436 (7)	0.0495 (8)	0.0786 (10)	−0.0023 (6)	0.0356 (7)	−0.0141 (7)
O2	0.0402 (7)	0.0527 (8)	0.0661 (9)	−0.0004 (6)	0.0268 (7)	−0.0056 (7)
O3	0.0411 (7)	0.0745 (10)	0.0535 (9)	0.0083 (7)	0.0273 (7)	0.0160 (7)
O4	0.0454 (7)	0.0667 (9)	0.0497 (8)	0.0079 (6)	0.0314 (7)	0.0085 (7)
O5	0.0402 (6)	0.0380 (7)	0.0443 (7)	0.0035 (5)	0.0272 (6)	0.0019 (5)
O6	0.0693 (9)	0.0384 (8)	0.0581 (9)	0.0081 (7)	0.0349 (8)	0.0032 (6)
O7	0.0642 (9)	0.0797 (11)	0.0483 (9)	−0.0177 (8)	0.0375 (8)	−0.0059 (7)
V1	0.03825 (18)	0.0450 (2)	0.0438 (2)	−0.00289 (13)	0.02761 (16)	−0.00409 (14)
V2	0.03993 (18)	0.03744 (19)	0.0434 (2)	0.00687 (12)	0.02607 (15)	0.00462 (13)

### Geometric parameters (Å, °)

**Table:**

C1—N1	1.485 (3)	C11—C12	1.499 (3)
C1—C2	1.495 (3)	C11—H11A	0.9700
C1—H1A	0.9700	C11—H11B	0.9700
C1—H1B	0.9700	C12—H12A	0.9600
C2—H2A	0.9600	C12—H12B	0.9600
C2—H2B	0.9600	C12—H12C	0.9600
C2—H2C	0.9600	C13—N4	1.481 (3)
C3—N1	1.471 (3)	C13—C14	1.496 (3)
C3—C4	1.516 (3)	C13—H13A	0.9700
C3—H3A	0.9700	C13—H13B	0.9700
C3—H3B	0.9700	C14—H14A	0.9600
C4—H4A	0.9600	C14—H14B	0.9600
C4—H4B	0.9600	C14—H14C	0.9600
C4—H4C	0.9600	C15—N4	1.470 (2)
C5—N2	1.470 (3)	C15—C16	1.509 (3)
C5—C6	1.503 (4)	C15—H15A	0.9700
C5—H5A	0.9700	C15—H15B	0.9700
C5—H5B	0.9700	C16—H16A	0.9600
C6—H6A	0.9600	C16—H16B	0.9600
C6—H6B	0.9600	C16—H16C	0.9600
C6—H6C	0.9600	N1—O1	1.403 (2)
C7—N2	1.464 (3)	N1—V1	2.0906 (16)
C7—C8	1.509 (3)	N2—O2	1.413 (2)
C7—H7A	0.9700	N2—V1	2.0797 (16)
C7—H7B	0.9700	N3—O3	1.408 (2)
C8—H8A	0.9600	N3—V2	2.0751 (17)
C8—H8B	0.9600	N4—O4	1.408 (2)
C8—H8C	0.9600	N4—V2	2.1004 (16)
C9—N3	1.476 (3)	O1—V1	1.8726 (14)
C9—C10	1.511 (3)	O2—V1	1.8790 (14)
C9—H9A	0.9700	O3—V2	1.8761 (14)
C9—H9B	0.9700	O4—V2	1.8719 (13)
C10—H10A	0.9600	O5—V1	1.8139 (11)
C10—H10B	0.9600	O5—V2	1.8151 (12)
C10—H10C	0.9600	O6—V2	1.5970 (14)
C11—N3	1.474 (3)	O7—V1	1.6012 (15)
			
N1—C1—C2	113.14 (18)	C13—C14—H14A	109.5
N1—C1—H1A	109.0	C13—C14—H14B	109.5
C2—C1—H1A	109.0	H14A—C14—H14B	109.5
N1—C1—H1B	109.0	C13—C14—H14C	109.5
C2—C1—H1B	109.0	H14A—C14—H14C	109.5
H1A—C1—H1B	107.8	H14B—C14—H14C	109.5
C1—C2—H2A	109.5	N4—C15—C16	114.32 (17)
C1—C2—H2B	109.5	N4—C15—H15A	108.7
H2A—C2—H2B	109.5	C16—C15—H15A	108.7
C1—C2—H2C	109.5	N4—C15—H15B	108.7
H2A—C2—H2C	109.5	C16—C15—H15B	108.7
H2B—C2—H2C	109.5	H15A—C15—H15B	107.6
N1—C3—C4	113.69 (19)	C15—C16—H16A	109.5
N1—C3—H3A	108.8	C15—C16—H16B	109.5
C4—C3—H3A	108.8	H16A—C16—H16B	109.5
N1—C3—H3B	108.8	C15—C16—H16C	109.5
C4—C3—H3B	108.8	H16A—C16—H16C	109.5
H3A—C3—H3B	107.7	H16B—C16—H16C	109.5
C3—C4—H4A	109.5	O1—N1—C3	110.38 (16)
C3—C4—H4B	109.5	O1—N1—C1	109.45 (14)
H4A—C4—H4B	109.5	C3—N1—C1	115.00 (16)
C3—C4—H4C	109.5	O1—N1—V1	61.13 (8)
H4A—C4—H4C	109.5	C3—N1—V1	121.56 (12)
H4B—C4—H4C	109.5	C1—N1—V1	122.23 (13)
N2—C5—C6	112.29 (19)	O2—N2—C7	111.01 (16)
N2—C5—H5A	109.1	O2—N2—C5	109.19 (15)
C6—C5—H5A	109.1	C7—N2—C5	116.45 (17)
N2—C5—H5B	109.1	O2—N2—V1	61.65 (8)
C6—C5—H5B	109.1	C7—N2—V1	120.83 (12)
H5A—C5—H5B	107.9	C5—N2—V1	121.08 (14)
C5—C6—H6A	109.5	O3—N3—C11	110.43 (15)
C5—C6—H6B	109.5	O3—N3—C9	110.55 (16)
H6A—C6—H6B	109.5	C11—N3—C9	116.07 (17)
C5—C6—H6C	109.5	O3—N3—V2	61.72 (8)
H6A—C6—H6C	109.5	C11—N3—V2	121.14 (14)
H6B—C6—H6C	109.5	C9—N3—V2	120.94 (13)
N2—C7—C8	114.75 (19)	O4—N4—C15	110.75 (15)
N2—C7—H7A	108.6	O4—N4—C13	109.80 (14)
C8—C7—H7A	108.6	C15—N4—C13	115.23 (17)
N2—C7—H7B	108.6	O4—N4—V2	60.75 (8)
C8—C7—H7B	108.6	C15—N4—V2	121.92 (12)
H7A—C7—H7B	107.6	C13—N4—V2	121.50 (14)
C7—C8—H8A	109.5	N1—O1—V1	77.86 (9)
C7—C8—H8B	109.5	N2—O2—V1	76.92 (9)
H8A—C8—H8B	109.5	N3—O3—V2	76.91 (9)
C7—C8—H8C	109.5	N4—O4—V2	78.23 (8)
H8A—C8—H8C	109.5	V1—O5—V2	154.12 (8)
H8B—C8—H8C	109.5	O7—V1—O5	118.13 (7)
N3—C9—C10	114.9 (2)	O7—V1—O1	107.65 (8)
N3—C9—H9A	108.5	O5—V1—O1	116.89 (6)
C10—C9—H9A	108.5	O7—V1—O2	109.80 (7)
N3—C9—H9B	108.6	O5—V1—O2	115.71 (6)
C10—C9—H9B	108.5	O1—V1—O2	83.41 (6)
H9A—C9—H9B	107.5	O7—V1—N2	94.45 (7)
C9—C10—H10A	109.5	O5—V1—N2	93.29 (6)
C9—C10—H10B	109.5	O1—V1—N2	124.84 (6)
H10A—C10—H10B	109.5	O2—V1—N2	41.43 (6)
C9—C10—H10C	109.5	O7—V1—N1	94.77 (8)
H10A—C10—H10C	109.5	O5—V1—N1	92.39 (6)
H10B—C10—H10C	109.5	O1—V1—N1	41.01 (6)
N3—C11—C12	112.42 (18)	O2—V1—N1	124.23 (6)
N3—C11—H11A	109.1	N2—V1—N1	165.33 (7)
C12—C11—H11A	109.1	O6—V2—O5	117.86 (7)
N3—C11—H11B	109.1	O6—V2—O4	109.61 (7)
C12—C11—H11B	109.1	O5—V2—O4	115.35 (6)
H11A—C11—H11B	107.9	O6—V2—O3	107.91 (7)
C11—C12—H12A	109.5	O5—V2—O3	117.74 (6)
C11—C12—H12B	109.5	O4—V2—O3	83.15 (6)
H12A—C12—H12B	109.5	O6—V2—N3	95.01 (7)
C11—C12—H12C	109.5	O5—V2—N3	93.08 (6)
H12A—C12—H12C	109.5	O4—V2—N3	124.33 (6)
H12B—C12—H12C	109.5	O3—V2—N3	41.37 (6)
N4—C13—C14	113.11 (18)	O6—V2—N4	94.26 (7)
N4—C13—H13A	109.0	O5—V2—N4	92.92 (6)
C14—C13—H13A	109.0	O4—V2—N4	41.02 (6)
N4—C13—H13B	109.0	O3—V2—N4	124.18 (6)
C14—C13—H13B	109.0	N3—V2—N4	165.06 (6)
H13A—C13—H13B	107.8		

### Table 1 Comparison of bond lengths (Å) and angles (°) between the previous determination (Saussine *et al.*, 1980) and the current study

**Table:**

Bond lengths	Reported	This work	Bond angles	Reported	This work
V1—N1	2.079 (4)	2.0906 (16)	O1—V1—N1	41.1 (1)	41.01 (6)
V1—N2	2.061 (4)	2.0797 (16)	O1—V1—O2	83.4 (1)	83.41 (6)
V1—O1	1.851 (3)	1.8726 (14)	O3—V2—O4	83.3 (1)	83.15 (6)
V1—O2	1.873 (3)	1.8790 (14)	O5—V1—O7	117.5 (1)	118.13 (7)
V1—O5	1.805 (3)	1.8139 (11)	N1—V1—N2	165.5 (1)	165.33 (7)
V1—O7	1.599 (3)	1.6012 (15)	V1—O5—V2	154.3 (1)	154.12 (8)
O1—N1	1.398 (5)	1.403 (2)	O5—V2—O6	117.6 (1)	117.86 (7)
O2—N2	1.400 (5)	1.413 (2)	O2—V1—N2	41.3 (1)	41.43 (6)
O3—N3	1.409 (5)	1.408 (2)	O3—V2—N3	41.2 (1)	41.37 (6)
O4—N4	1.402 (5)	1.408 (2)	O4—V2—N4	41.2 (1)	41.02 (6)
